# The Synergistic Effect of TRPV1 on Oxidative Stress-Induced Autophagy and Apoptosis in Microglia

**DOI:** 10.1155/2021/7955791

**Published:** 2021-07-15

**Authors:** Tingting Huang, Yao Lin, Qiongyi Pang, Weimin Shen, Xiang Chen, Fengxia Tu

**Affiliations:** Yuying Children's Hospital of Wenzhou Medical University, No. 109, Xueyuan West Road, Wenzhou, Zhejiang, China

## Abstract

Stroke mostly including ischemic stroke is the second leading mortality and disability worldwide. Oxidative stress injury occurred during ischemic stroke treatment generally. A high amount of reactive oxygen species (ROS) is involved in oxidative stress induction. Transient receptor potential vanilloid 1 (TRPV1) has been shown to regulate oxidative stress and apoptosis in microglia; however, the detailed mechanisms remain unclear. We aimed to explore whether autophagy-regulated oxidative stress and apoptosis are associated with TRPV1. The model of oxygen and glucose deprivation (OGD/R) in microglia was established. The siRNA of Atg5 and inhibitors and agonists of both autophagy and TRPV1 were involved in our study. Autophagy-related markers Atg5, LC3II/LC3I, and Beclin-1 were measured, and the autophagosome was observed under a transmission electron microscope (TEM). Caspase 3 was detected using ELISA. ROS and JC-1 were detected using flow cytometry. Apoptosis was observed by TUNEL. The results indicated that oxidative stress-induced injury and apoptosis may be impeded by the increasing autophagy, and TRPV1 inhibition could suppress the OGD/R-induced autophagy of microglia. However, the effect of TRPV1's inhibitor on oxidative stress and apoptosis was not obvious when the autophagy was blocked. These findings suggested that TRPV1 may exhibit antioxidative and antiapoptosis effect on OGD/R-induced microglia. However, the experimental results do not fully demonstrate that the TRPV1-mediated antioxidative and antiapoptosis effect is through the affecting autophagy entirely.

## 1. Introduction

Ischemic stroke accounted for 87% of stroke [1], due to which paralysis and death occur leadingly worldwide. Moreover, the rate of disability and mortality ranks second in China [2]. Cerebral ischemia-reperfusion injury is often accompanied by the stroke treatment [3].

Microglia are brain resident macrophages, and oxidative stress-induced autophagy and apoptosis of microglia play an important role in cerebral ischemia-reperfusion injury [4]. As a voltage-gated calcium channel, transient receptor potential vanilloid 1 (TRPV1) is confirmed to be widely expressed in the brain. According to previous studies, TRPV1 is naturally expressed in microglia endomembrane to be mainly permeable to Ca^2+^. In the brain, TRPV1 can inhibit inflammatory response in the brain and acts importantly in microglia activation [5–7]. However, it has been confirmed that overactivated TRPV1 may facilitate microglia-induced inflammation [8]. As reported in a previous study, activation of TRPV1 by CuS could attenuate OxLDL-induced autophagy impairment through activating the Ca^2+^-AMPK pathway as well as expediting cholesterol efflux ABCA1-dependentlly [9]. Apoptosis leads to cell death, and the role of accompanied autophagy is more complicated. Microglia activation is related to the apoptosis of microglia under oxidative stress injury [10]. Autophagy is a mechanism for either protecting or killing stressed cells [11]. The balance of signaling pathways between apoptosis and proliferation may be modified by TRPV1 [12]. The role of TRPV1 in oxidative stress-induced autophagy and apoptosis in microglia remains unclear, and further research is needed. An in-depth understanding of the specific role of autophagy and apoptosis in response to cerebral ischemia injury mediated by microglia may provide a new therapeutic target for the prevention and treatment of cerebral ischemia-reperfusion injury.

In the present study, we investigated the role of TRPV1 in cerebral ischemia-reperfusion injury through establishing a ischemic stroke model of oxygen and glucose depreciation/reoxygenation (OGD/R) in vitro as well as the effect of TRPV1 in ischemia-reperfusion injury, and OGD/R-induced autophagy along with apoptosis in microglia was also explored in our study. Our results involve the effect of TRPV1 in the autophagy and apoptosis of microglia induced by oxidative stress injury, and the elucidation of its mechanism would illuminate the way to ischemic stroke prevention and treatment.

## 2. Materials and Methods

### 2.1. Microglia Isolation and Purification

Primary microglia were isolated from the cortical tissue of a neonatal rat and cultured according to a previous study [13]. In brief, the cortical tissue was digested using trypsin with collagenase, then resuspended in culture, and incubated at 37°C in a 5% CO_2_ atmosphere. The microglia were next purified by a rotary shaker after 9 days incubation followed by resuspension in DMEM with poly-L-lysine to obtain primary microglia for subsequent experiments.

### 2.2. OGD/R Model of Microglia and Treatment

Microglia were exposed to oxygen deprivation as 1% O_2_ + 5% CO_2_ + 94% N_2_ for 6 h in glucose-free culture and then reoxygenated for 24 h. After hypoxia, the cells were reoxygenated for 24 h in the complete medium.

10 mmol/ml of autophagy inhibitor 3MA (Meilunbio, China), 200 nmol/ml of autophagy agonist RAPA (Meilunbio, China), 1 *μ*mol/ml of TRPV1 agonist Cap (Meilunbio, China), and TRPV1 inhibitor iRTX (Meilunbio, China) were chosen to pretreat microglia just before OGD/R.

### 2.3. siRNA Transfection

Microglia were transfected with Atg5 siRNA (siAtg5), negative control (NC) siRNA assisted by transfection reagent (Roche). Microglia were incubated with Atg5 siRNA or NC with transfection reagent in 12-well plates for 48 h and then used for subsequent experiments.

### 2.4. Flow Cytometry (FCM)

For ROS detection, after OGD/R with/without treatment, the microglia was incubated with 1 ml of medium including diluted DCFH-DA (ROS) (1 : 1000) (Beyotime, China) at 37°C for 20 minutes. The cells were then washed three times with serum-free cell culture medium and collected to assay the ROS level using flow cytometry (CytoFLEX, Becman, USA).

For the JC-1 assay, 6 × 10^5^ cells in 0.5 ml medium were incubated with 0.5 ml JC-1 staining working solution at 37°C for 20 minutes. After incubation, the cells were centrifuged (600 × g) at 4°C for 3-4 minutes to remove the supernatant. The cells were then washed twice using the JC-1 staining buffer (1X) followed by resuspension with an appropriate amount of the JC-1 staining buffer (1X). JC-1 was detected using flow cytometry and analyzed by the CytExpert software. The JC-1 assay reagent was purchased from Beyotime (China).

### 2.5. Transmission Electron Microscopy

To observe the autophagy, microglia were firstly fixed with glutaraldehyde (2.5%) and osmium acid (1%). Then EM KMR3 was used to embed the cells, and slicing was made using an ultrathin microtome (EM UC7). The autophagosome was observed and photographed using a transmission electron microscope (JEM-1400PLUS).

### 2.6. Western Blotting

The proteins of Atg5, LC3II/LC3I, and Beclin-1 were measured using western blotting according to a previous study [14]. After OGD/R with/without treatment, microglia were collected and then lysed for proteins extraction. Equal amounts total protein were analyzed using western blotting. The primary antibodies to *β*-actin (1 : 1000, Abclonal, China), LC3II/I (1 : 250, Abclonal, China), Atg5 (1 : 250, Abclonal, China), and Beclin-1 (1 : 1000, Abclonal, China) were used.

### 2.7. TUNEL Staining

Smears of microglia were fixed with 4% paraformaldehyde in 0.01 M PBS (pH 7.0-7.6) at room temperature for 45 minutes and then washed twice using both 0.01 M PBS and distilled water for 2 minutes each. The specimens were digested using proteinase in TBS (0.01 M, 1 : 200) for 10 minutes at 37°C and then washed three times for 2 minutes each. The smears were kept moist using labeling buffer including TDT, in turn with the DIG-D-UTP-labeling buffer at 37°C for 2 hours in a wet box. After washing three times by TBS (0.01 M) for 2 minutes each, the blocking solution was added to the smears at room temperature for 30 min, and then the biotinylated anti-digoxin antibody was added followed by reacting at 37°C for 30 min in a wet box. The smears were washed three times by TBS (0.01 M) for 2 minutes each and reacted with the SABC antibody (1 : 100) at 37°C for 30 minutes and then washed four times for 5 minutes each. The nucleus was stained using the DAPI staining solution (Beyotime, China). After sealing using the antifluorescent decaying tablet, the smears' photos were taken using the fluorescence microscopic imaging system (Guangzhou Mingmei, China).

### 2.8. ELISA Assay

The level of caspase 3 was assayed using ELISA kits (Cloud-Clone, China) according to the kits' instructions.

## 3. Statistical Analysis

The data was statistically analyzed using the SPSS 20.0 statistical software. Student's two-tailed *t*-test was used to compare the two groups, and one-way analysis of variance (ANOVA) was used for the statistical analysis for three or more groups. The data were obtained from at least three independent experiments and expressed as mean ± SD. When the *p* < 0.05, the difference was considered statistically significant.

## 4. Results

### 4.1. Autophagy Negatively Correlated with ROS Level in OGD/R Microglia

**Figure 1:**
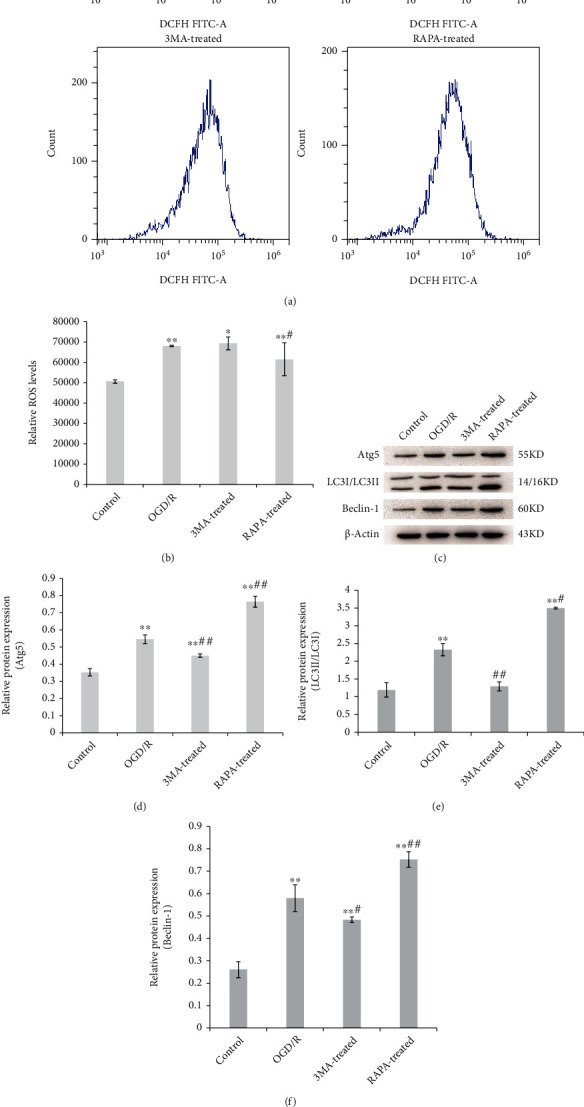
The relation of oxidative stress and autophagy in microglia. The ROS level, autophagy-related proteins, and autophagolysosomes were measured in microglia and OGD/R-induced microglia, and the autophagy inhibitor 3MA and autophagy agonist RAPA were involved. (a, b) ROS level was detected using flow cytometry. (c) Autophagy-related proteins were detected using western blotting including Atg5 (d), LC3I/II (e), and Beclin-1 (f). (g) Autophagolysosomes (red arrow) were observed using transmission electron microscope. The data was given as mean ± SD, *n* = 3. ^∗∗^*p* < 0.01 compared with microglia in culture (control). ^#^*p* < 0.05 and ^##^*p* < 0.01 compared with microglia on glucose depreciation/reoxygenation (OGD/R) culture condition (OGD/R).

The level of ROS in microglia or OGD/R-induced cells with/without treatment of 3MA or RAPA. The results are shown in Figures 1(a) and 1(b), in which the level of ROS was significantly increased in OGD/R microglia compared with control cells (normal cells). Autophagy agonist RAPA decreased OGD/R-induced increase ROS significantly, although the ROS level was still higher than that of control cells. No significant difference was observed in the autophagy inhibitor 3MA-treated cells compared with control cells.

The expression of autophagy-related proteins Atg5, LC3II/LC3I, and Beclin-1 was indicated by the results of western blotting (Figures 1(c)–1(f)). All the autophagy-related proteins detected in the present study were significantly increased in OGD/R microglia compared with control cells, and 3MA inhibited, as well as RAPA promoted, the protein expression of Atg5, LC3II/LC3I, and Beclin-1 obviously. The results from the transmission electron microscopy also indicated that OGD/R-induced increased autophagosomes and 3MA suppressed while RAPA expedited autophagy. All the above results suggested that autophagy may negatively correlate with the ROS level in OGD/R microglia.

### 4.2. Apoptosis Associates with Autophagy in OGD/R Microglia

**Figure 2:**
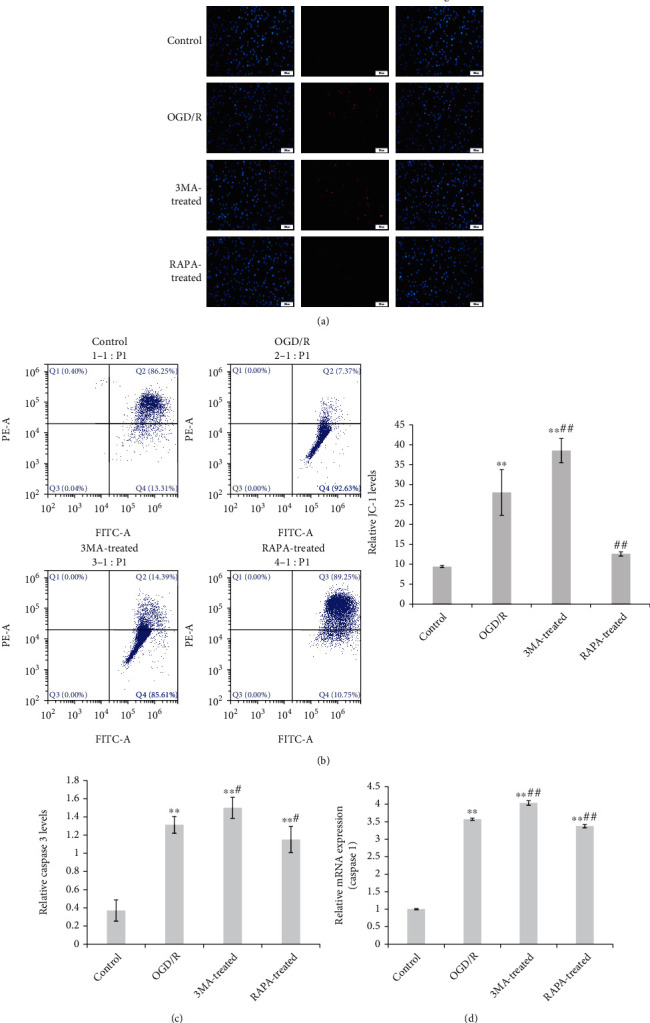
Oxidative stress-induced apoptosis accompanied with autophagy in microglia. Apoptosis was assayed in microglia and OGD/R-induced microglia, and the autophagy inhibitor 3MA and autophagy agonist RAPA were involved. Apoptosis was measured using both TUNEL staining (×800) (a) and JC-1 assay by flow cytometry (b). The apoptosis-related protein of caspase 3 was also detected using the ELISA kit (c). The mRNA expression of caspase 1 was detected using qPCR (d). The data was given as mean ± SD, *n* = 3. ^∗∗^*p* < 0.01 compared with microglia in culture (control). ^#^*p* < 0.05 and ^##^*p* < 0.01 compared with microglia on glucose depreciation/reoxygenation (OGD/R) culture condition (OGD/R).

Apoptosis of microglia or OGD/R-induced cells with/without treatment of 3MA or RAPA was measured using both TUNEL staining and multimer-mono conversion of JC-1. As shown in Figure 2(a), apoptosis of microglia exhibited by TUNEL staining positive cells was higher in OGD/R microglia than control cells, 3MA-promoted as well as RAPA-inhibited apoptosis of OGD/R microglia. The results of the JC-1 assay were similar to that of TUNEL staining. The ratio of green to red indicated mono/multimer of JC-1 that was significantly increased in OGD/R microglia, and 3MA aggravated while RAPA attenuated OGD/R induction to JC-1 transformation (Figures 2(b) and 2(c)). The level of caspase 3, an apoptosis-related protein, is significantly increased in OGD/R microglia. 3MA promoted as well as RAPA suppressed the caspase 3 level in OGD/R microglia (Figure 2(d)). The results confirmed that 3MA- or RAPA-mediated autophagy diversification was associated with apoptosis in OGD/R microglia.

### 4.3. Atg5-Mediated Autophagy Combined with TRPV1 Correlated with the ROS Level in OGD/R Microglia

**Figure 3:**
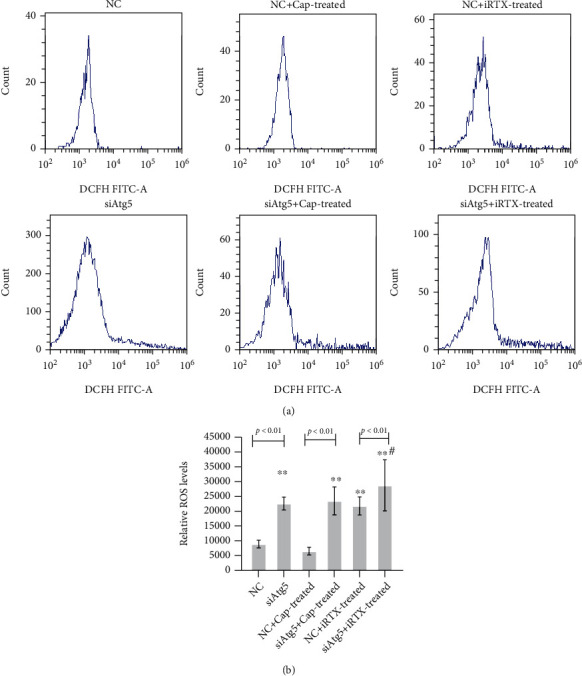
TRPV1 accompanied with Atg5-mediated autophagy in oxidative stress-induced microglia. The ROS level was measured in OGD/R microglia, and an autophagy-related protein of siRNA-Atg5 (siAtg5) along with negative (NC) siRNA-transfected cells, TRPV1 agonist Cap, and TRPV1 inhibitor iRTX was involved. (a) ROS level was detected using flow cytometry and analyzed (b). The data was given as mean ± SD, *n* = 3. ^∗∗^*p* < 0.01 compared with microglia in culture (control). ^#^*p* < 0.05 compared with microglia on glucose depreciation/reoxygenation (OGD/R) culture condition (OGD/R).

Due to the increasing expression of Atg5, an autophagy-related protein, in OGD/R microglia, the siRNA of Atg5 and TRPV1 agonist Cap along with TRPV1 inhibitor iRTX were involved in the following experiments. It is shown in Figure 3 that downregulated Atg5 expression induced ROS increasing in OGD/R microglia, and iRTX enhanced the ROS level both in negative control (NC) siRNA and in the siRNA of Atg5- (siAtg5-) transfected OGD/R microglia. The effect of Cap was slight on NC or siAtg5-transfected OGD/R microglia individually compared with that without Cap treatment cells. TRPV1 activation may antagonize the effect of Atg5-mediated autophagy on oxidative stress to microglia.

### 4.4. TRPV1 Associated with Apoptosis and Atg5 Mediated Autophagy in OGD/R Microglia

**Figure 4:**
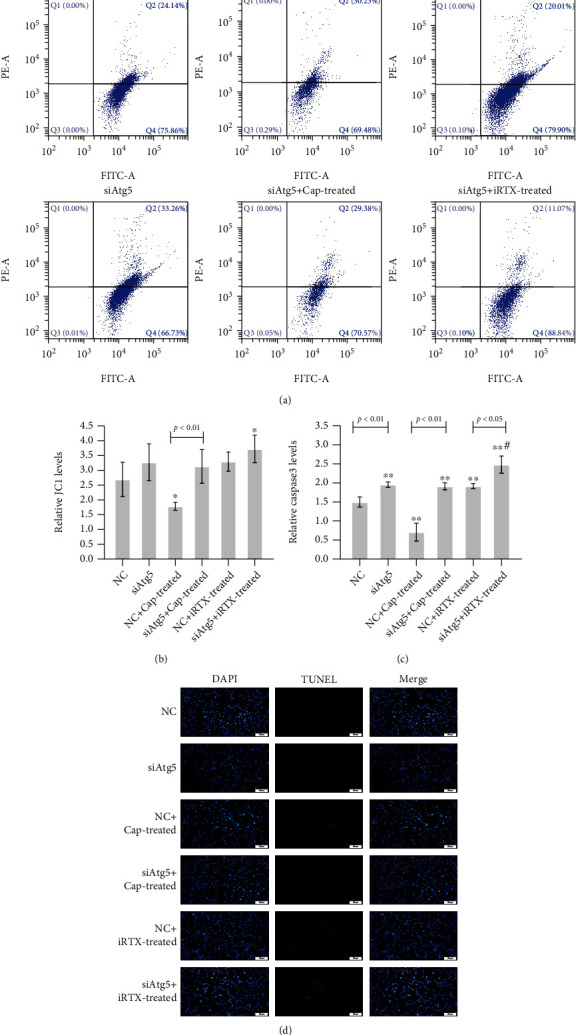
TRPV1 and apoptosis, Atg5-mediated autophagy in OGD/R microglia. Apoptosis was assayed in OGD/R microglia, and an autophagy-related protein of siRNA-Atg5 (siAtg5) along with negative (NC) siRNA-transfected cells, TRPV1 agonist Cap, and TRPV1 inhibitor iRTX was involved. (a, b) JC-1 was assayed by flow cytometry and analyzed. (c) The level of caspase 3 was measured using the ELISA kit. (d) Apoptosis was assayed using TUNEL staining. The data was given as mean ± SD, *n* = 3. ^∗^*p* < 0.05 and ^∗∗^*p* < 0.01 compared with microglia in culture (control). ^#^*p* < 0.05 compared with microglia on glucose depreciation/reoxygenation (OGD/R) culture condition (OGD/R).

Apoptosis was measured using the JC-1 assay, TUNEL staining, and caspase 3 level in OGD/R microglia transfected with NC/siAtg5 with/without treatment of Cap or iRTX. The ratio of green to red indicated that the mono/multimer of JC-1 was significantly increased in siAtg5-transfected OGD/R microglia compared with NC cells (Figures 4(a) and 4(b)). iRTX aggravated as well as Cap attenuated JC-1 transformation in both NC and siAtg5-transfected OGD/R microglia. The results of TUNEL staining was similar to the JC-1 assay that siAtg5 inducing apoptosis increase in OGD/R microglia and iRTX promoted apoptosis further in siAtg5-transfected OGD/R microglia, as well as inducing increase apoptosis in NC cells significantly (Figure 4(d)). The effect of Cap on apoptosis in both NC and siAtg5-transfected OGD/R microglia was fewer than that in no treated NC or siAtg5-transfected cells. The level of caspase 3 obtained from ELISA indicated that siAtg5 transfection induced increasing caspase 3 level which was increased by iRTX treatment (Figure 4(c)). It was worth noting that there was no significant difference among siAtg5, siAtg5 with Cap treatment, and NC with iRTX treatment OGD/R microglia (*p* > 0.05).

## 5. Discussion

Stroke is the second leading cause of mortality and accounts for 9% of deaths worldwide [15]. Ischemic stroke is the most common kind of stroke [16]. Restoring blood supply flow in acute ischemic stroke remains the most important available treatment to acute ischemic stroke, during which oxidative stress might occur [17]. However, that treatment involved in reperfusion will lead to a highly harmful ROS production and generate oxidative stress to be responsible for most of the ischemia-reperfusion injury and thus causing damage to cerebral tissue [17, 18]. As one of markers in oxidative stress-induced brain injury, ROS is complicated in brain injury after ischemic stroke [15]. It has been proven that the prompt increase of ROS production after acute ischemic stroke could immediately overwhelm the antioxidant defending to induce tissue damage further [19], whereas low levels of reactive oxygen species (ROS) are crucial for maintaining cancer stem cells (CSCs) and their ability to resist therapy [20]. As reported in previous studies, ROS can do great damage to cellular macromolecules that can lead to autophagy, apoptosis, and necrosis of brain cells such as neurons, astrocytes, or microglia [15, 21–23].

Microglia are considered brain macrophages derived from yolk sac progenitor cells. Microglia, as the innate immune cells in the brain, account for 30%-50% of the infiltrating cells in the glioma microenvironment. In addition, studies have shown that the grade of glioma is proportional to the number of infiltrating “M2”-type microglia in the microenvironment. Therefore, glioma-associated microglia and macrophages are also one of the research hotspots in the microenvironment of glioma [24]. Microglia can be activated and recruited to the lesion site within a few hours. Microglia activation is traditionally thought to play a detrimental role in ischemic stroke, and suppressing microglia activation could attenuate ischemia-induced brain damage [4]. Increasing ROS frequently emerges accompanied with microglia activation, and elevated ROS in microglia leads to activation of inflammatory and cell death pathways as autophagy, apoptosis, and necrosis [25, 26]. When autophagy occurs, the components of cells including macroproteins or even whole organelles are sequestered into lysosomes for degradation [27]. Autophagy can be initiated most notably by nutrient deprivation similar to OGD/R to microglia [11]. In the present study, we established the oxidative stress injury model (OGD/R) in microglia at first. Both ROS level and autophagy involved in OGD/R microglia were also investigated. Our results showed that oxidative stress-related ROS level tended to be negatively correlated with autophagy in OGD/R microglia, and autophagy agonist could attenuate OGD/R-induced increasing ROS level in microglia, which suggested that autophagy in the early stage of oxidative stress may suppress ROS elevation.

Autophagy not only is used to recycle cellular components frequently but also leads to cellular destruction to remove senescent cells from aged and lesion tissues induced by oxidative stress [27–29]. Autophagy tends to serve as both protecting and killing stressed cells including apoptosis [27, 30]. It has been hypothesized that in early stages of tumorigenesis, autophagy has a tumor-suppressor role while, in more advanced stages, it may represent a survival mechanism of neoplastic cells in response to stress [31]. We then explored the role of autophagy in correlation with oxidative stress-related apoptosis in microglia. The apoptosis results from JC-1, TUNEL staining, and apoptosis-related protein of caspase 3 indicated that enhancing the autophagy by RAPA attenuated OGD/R-induced apoptosis in microglia. Our results suggested that enhanced autophagy contributes to reducing oxidative stress-related apoptosis in microglia. Considering the autophagy-related protein of Atg5 was significantly increased in OGD/R microglia and obviously correlated with ROS level and apoptosis negatively in oxidative stress-induced cells, the siRNA of Atg5 was used in the following study.

Intracellular concentration of Ca^2+^ is implicated to determinate cell fate. Depending on the driving forces, ions including Ca^2+^ can enter or exit the cell through ion channels in the membrane to maintain ion balance, which correlates with cell fate closely. TRPV1, expressed widely in brain, is noted as a ligand-gated ion channel to facilitate transmembrane entry of Ca^2+^ and Na^+^, which may modify a delicate balance in apoptotic signaling pathways [12]. The DNA fragmentation and condensation, caspase activation, influx and efflux of Ca^2+^ in the cytosol, and so on are involved in the TRPV1-related pathway in apoptosis. It was also found that ROS could activate TRPV1-delayed onset muscle soreness [32]; however, TRPV1 showed a ROS-independent activation in HEK293 or vagal neurons [33]. Although activation of TRPV1 is involved in autophagy impairment [9], the role of TRPV1 in both oxidative stress-induced autophagy and apoptosis in microglia remains unclear. Then, the TRPV1 agonist Cap and TRPV1 inhibitor iRTX accompanied with siRNA of an autophagy-related protein as Atg5 were involved in the following study. Our results indicated that ROS was increased significantly in low Atg5 expression of OGD/R microglia, while iRTX promoted the ROS level of all the OGD/R microglia with/without the siRNA of Atg5. The results of apoptosis assay from JC-1 assay, TUNEL staining, and caspase 3 were similar to ROS detection, in which low expression of Atg5 promoted apoptosis and iRTX aggravated apoptosis further in OGD/R microglia. Cap individually affected the ROS level in NC or siAtg5-transfected OGD/R microglia slightly. Nevertheless, Cap had fewer effect on apoptosis in both NC and siAtg5-transfected OGD/R microglia than that in no treated NC or siAtg5-transfected cells.

## 6. Conclusion

In conclusion, our results suggested that increasing autophagy occurred during oxidative stress-induced injury mediated by ROS to protect microglia from oxidative stress-induced apoptosis. Inhibiting TRPV1 may antagonize the effect of Atg5-mediated autophagy and aggravate the apoptosis in microglia induced by oxidative stress.

## Data Availability

All data of this study are available on request from the corresponding author.

## Abbreviations

OGD/R: Oxygen-glucose deprivation/reoxygenation
DMSO: Dimethyl sulfoxide
TRPV1: Transient receptor potential vanilloid 1
ROS: Reactive oxygen species.
